# Concurrent Onset of Osimertinib-Induced Heart Failure and Metronidazole-Induced Encephalopathy During Brain Abscess Treatment: A Report of a Rare Case

**DOI:** 10.7759/cureus.75079

**Published:** 2024-12-04

**Authors:** Emi Mikami, Kosuke Hashimoto, Kyoichi Kaira, Atsuto Mouri, Yu Miura, Ayako Shiono, Ou Yamaguchi, Hisao Imai, Hiroshi Kagamu

**Affiliations:** 1 Clinical Training Center, Saitama Medical University International Medical Center, Hidaka, JPN; 2 Respiratory Medicine, Saitama Medical University International Medical Center, Hidaka, JPN

**Keywords:** encephalopathy, heart failure, metronidazole, osimertinib, weight loss

## Abstract

We report a rare case of concurrent onset of osimertinib-induced heart failure and metronidazole-induced encephalopathy during treatment of a brain abscess. A 78-year-old female with lung adenocarcinoma presented with neurological symptoms and was diagnosed with a brain abscess. During treatment, she developed heart failure and encephalopathy, linked to osimertinib and metronidazole, respectively. Following the discontinuation of these drugs, her symptoms gradually improved. This case underscores the need for careful monitoring of adverse drug reactions, especially in patients with significant weight loss. Regular cardiac and neurological assessments are recommended to promptly identify and manage such events.

## Introduction

Osimertinib, a third-generation epidermal growth factor receptor (EGFR)-tyrosine kinase inhibitor, is commonly used to treat EGFR-mutant non-small cell lung cancer. It is associated with adverse cardiac events, including heart failure, with an incidence of up to 6.1% [1]. The mechanism involves the inhibition of cardiac ion channels, leading to prolonged PR and QT intervals, QRS complex prolongation, and potentially resulting in cardiomyopathy and heart failure [2]. Additionally, patients with low body weight have an increased risk of adverse events [3], as lower body weight can lead to higher plasma concentrations of the drug, increasing toxicity risk.

Metronidazole, an antibiotic frequently used to treat anaerobic infections, can cause metronidazole-induced encephalopathy (MIE), a rare but serious central nervous system disorder. A systematic review identified 136 cases of MIE in 112 studies. The proposed mechanisms of MIE include oxidative stress and mitochondrial dysfunction, which disrupt central nervous system metabolic processes [4]. Furthermore, patients with low body weight have a high risk of adverse neurological events [5] due to altered pharmacokinetics and pharmacodynamics [6].

In this case, the patient experienced significant weight loss during the treatment of a brain abscess. This weight loss may have contributed to the increased risk of adverse events from both osimertinib and metronidazole. This case report aims to highlight the frequency of these complications when using these treatments and discuss the impact of weight loss on these adverse events. The combination of these two drugs in a patient with a brain abscess and significant weight loss is underexplored in the literature, emphasizing the novelty of this case. Studies addressing polypharmacy and adverse event monitoring in cancer patients further support the need for careful drug management and monitoring to prevent severe adverse events.

## Case presentation

**Figure 1:**
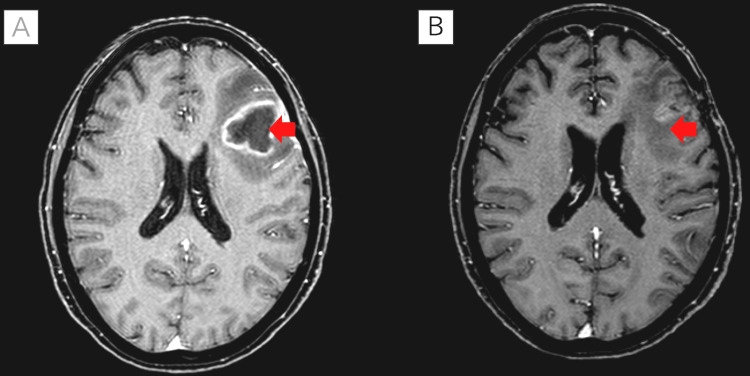
MRI of brain abscess formation and post-treatment changes (red arrows) (A) This MRI scan shows the formation of a brain abscess, indicated by a ring-enhancing lesion. (B) This MRI scan shows the area after the operation for the brain abscess, highlighting the resulting scarring.

A 78-year-old female with EGFR-mutant lung adenocarcinoma had been treated with osimertinib for 38 months when she developed short-term memory impairment and motor coordination deficits, raising the suspicion of brain metastasis. Magnetic resonance imaging (MRI) revealed a brain abscess. She was transferred to another hospital, where she underwent surgical drainage of the abscess and was administered intravenous ceftriaxone and metronidazole postoperatively (Figure 1).

During the postoperative period, the patient experienced rapid weight loss from 43 to 34 kg, primarily due to nutritional challenges. After approximately 40 days of intravenous treatment, the patient suddenly developed dyspnea and worsening neurological symptoms, which led to her transfer to our hospital. Chest radiography revealed cardiomegaly, and osimertinib-induced heart failure was considered. Consequently, osimertinib administration was discontinued, and furosemide (40 mg/day) was initiated.

**Figure 2:**
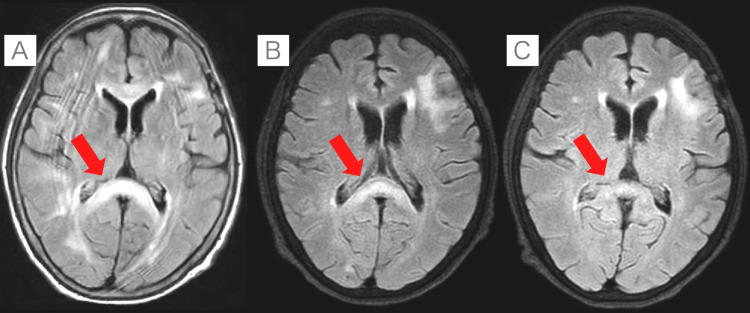
Temporal changes in MRI fluid-attenuated inversion recovery (FLAIR) images of metronidazole encephalopathy (red arrows) (A) MRI image showing a hyperintense lesion in the splenium of the corpus callosum consistent with metronidazole-induced encephalopathy. (B, C) Images taken on the 9th and 14th days after diagnosis, respectively. The hyperintense lesion in the splenium of the corpus callosum shows a gradual reduction over time.

Neurological examination revealed short-term memory impairment and motor coordination deficits but no other neurological deficits. Echocardiography was performed, revealing an ejection fraction (EF) of 17%. An MRI of the brain showed high signal intensity in the splenium of the corpus callosum, which was consistent with MIE. Follow-up scans on days 9 and 14 after discontinuation of metronidazole showed improvement (Figure 2).

The patient's neurological symptoms gradually improved, and her EF improved to 34%. She was discharged on postoperative day 84 with home care support. Further follow-up for the management of her lung adenocarcinoma and brain abscess was scheduled.

## Discussion

This case emphasizes the importance of focusing on body weight and recognizing and managing drug-induced adverse effects. The risk of osimertinib-induced heart failure and MIE increases in patients with low body weight [3,5]. The patient’s significant weight loss likely contributed to the increased blood concentrations of both drugs, leading to the observed adverse effects.

Furthermore, the onset of heart failure can affect the absorption, distribution, and excretion of drugs in the liver and kidneys [7]. Osimertinib-induced heart failure may have altered the pharmacokinetics of metronidazole, increasing its blood concentration and contributing to encephalopathy development [8,9].

MIE is a rare but serious condition that typically presents with symptoms such as dysarthria, gait instability, limb dyscoordination, and altered mental status. MRI findings often show symmetrical hyperintense lesions on T2/fluid-attenuated inversion recovery in the dentate nuclei. MIE usually improves significantly after discontinuation of metronidazole. Elevated blood concentrations of metronidazole can lead to toxicity, and measuring these levels can help confirm the diagnosis of encephalopathy and other complications [8,9].

Although we did not measure the blood concentration of metronidazole in this case, this should be considered in future studies. In patients with significant weight loss or heart failure, attention should be paid to the increased risk of drug-induced adverse effects. The idea that osimertinib-induced heart failure might alter the pharmacokinetics of metronidazole is a plausible hypothesis. Heart failure can lead to changes in drug absorption, distribution, and excretion by affecting liver and kidney function. Reduced cardiac output in heart failure can decrease hepatic blood flow, impairing drug metabolism. Additionally, renal clearance may be affected due to reduced renal perfusion and altered glomerular filtration rate [8,9]. Monitoring blood concentrations of metronidazole is appropriate for diagnosing MIE and adjusting the dose. Elevated levels can indicate toxicity and necessitate dose adjustments [4].

Specific strategies for mitigating these risks include adjusting drug doses in patients with significant weight loss and implementing monitoring strategies such as echocardiograms and renal function tests.

Regular monitoring can help prevent complications and ensure patient safety. Weight loss influences drug pharmacokinetics by altering drug distribution and clearance. 

## Conclusions

This case highlights the impact of significant weight loss on drug pharmacokinetics and the associated risk of adverse drug reactions during polypharmacy. The patient’s severe weight loss likely increased blood concentrations of osimertinib and metronidazole, contributing to heart failure and encephalopathy. Early recognition and appropriate management, considering factors like weight loss and heart failure, are essential for improving outcomes. To mitigate these risks, it is crucial to emphasize specific monitoring strategies and recommended changes to treatment in patients with similar risk factors. Regular assessment and adjustment of medication doses based on the patient's weight changes are necessary to prevent toxicity. Patients experiencing significant weight loss should have their drug dosages evaluated more frequently. Frequent monitoring of drug levels, clinical symptoms, and laboratory markers is essential to detect adverse reactions early. Tools such as echocardiograms and renal function tests should be used to track heart and kidney functions. Furthermore, advocating for clinical trials and cohort studies to assess the impact of weight loss on drug pharmacokinetics in patients with cancer or those undergoing complex treatments can provide valuable data to inform future treatment guidelines and improve patient safety. By adopting these strategies, healthcare providers can better manage the complexities of polypharmacy in patients experiencing significant weight loss, ultimately improving patient outcomes.
